# Assessment of Residual Oxidative Stress in Patients with Well-Controlled Hypertension: A Pilot Cross-Sectional Study

**DOI:** 10.3390/medsci13040292

**Published:** 2025-11-28

**Authors:** Wuthichai Preechakul, Putcharawipa Maneesai, Poungrat Pakdeechote, Weerapon Sangartit, Metee Iampanichakul, Kittisak Sawanyawisuth, Somchai Ruangwannasak, Sittichai Khamsai

**Affiliations:** 1 Department of Physiology, Faculty of Medicine, Khon Kaen University, Khon Kaen 40002, Thailand; wuthichai.p@kkumail.com (W.P.); putcma@kku.ac.th (P.M.); ppoung@kku.ac.th (P.P.); weerasan@kku.ac.th (W.S.); meteiam@kku.ac.th (M.I.); 2 Department of Medicine, Faculty of Medicine, Khon Kaen University, Khon Kaen 40002, Thailand; kittisak@kku.ac.th; 3 Department of Surgery, Faculty of Medicine, Khon Kaen University, Khon Kaen 40002, Thailand; somcru@kku.ac.th

**Keywords:** ankle–brachial index, biomarkers, well-controlled hypertension, oxidative stress, residual risk

## Abstract

**Background/Objectives:** Although hypertension is linked to oxidative stress, it remains unclear whether this pro-oxidant state persists after achieving recommended blood pressure (BP) control. This pilot study aimed to explore the presence of residual oxidative stress in patients with well-controlled hypertension compared to normotensive individuals. **Methods:** In this cross-sectional pilot study, 34 adults were enrolled: 20 normotensive controls and 14 patients with well-controlled hypertension (office BP < 140/90 mmHg). Macrovascular status was assessed by ankle–brachial index (ABI), and plasma concentrations of malondialdehyde (MDA), superoxide dismutase (SOD), catalase, tumor necrosis factor-alpha (TNF-α), and interleukin-6 (IL-6) were measured. Hypertensive patients were further stratified by median MDA levels for subgroup analysis. **Results:** Baseline characteristics, including BP, were similar between groups. However, patients with well-controlled hypertension exhibited significantly higher plasma MDA concentrations compared to normotensive controls (9.91 ± 6.07 vs. 4.73 ± 2.34 µmol/L, *p* = 0.008). In subgroup analysis, hypertensive patients with high MDA were significantly older (*p* = 0.03) and showed a trend towards higher systolic BP (*p* = 0.05) compared to those with low MDA. No significant differences were observed in SOD or catalase activity, ABI, or inflammatory markers (all *p* > 0.05). **Conclusions:** Residual oxidative stress—as reflected by increased plasma MDA—persists in patients with well-controlled hypertension. While this oxidative state appears broadly independent of BP when viewed as a whole, it is notably more pronounced in older patients and in those with systolic BP approaching the upper limit of the controlled range. These findings support the need for comprehensive, biomarker-based risk assessment and further investigation into targeted strategies for mitigating persistent redox imbalance in hypertension.

## 1. Introduction

Hypertension affects over one billion adults worldwide and remains a leading cause of cardiovascular morbidity and mortality [1,2]. International guidelines from major organizations emphasize achieving evidence-based blood pressure (BP) targets as the primary therapeutic objective to reduce cardiovascular risk [3,4]. Nevertheless, substantial evidence demonstrates that a significant proportion of patients experience major cardiovascular events despite achieving guideline-recommended BP levels, thereby highlighting persistent residual risk beyond BP normalization [5,6,7].

This residual risk suggests the presence of additional pathophysiological mechanisms, independent of hemodynamic stress, that continue to drive vascular injury in treated hypertension. Oxidative stress has emerged as a critical and multifactorial mediator in this context [8,9,10]. Excessive generation of reactive oxygen species (ROS)—particularly due to upregulation of vascular NADPH oxidases and compromised antioxidant defenses—impairs nitric oxide (NO) bioavailability, disrupts endothelial function, triggers vascular remodeling, and accelerates atherosclerosis progression [10,11,12]. In addition, emerging evidence implicates immune-mediated mechanisms, such as T cell–derived hydrogen peroxide, in sustaining ROS production even under pharmacological BP control [13,14].

From a biomarker perspective, malondialdehyde (MDA) is a well-established indicator of lipid peroxidation, while antioxidant enzymes, including superoxide dismutase (SOD) and catalase, reflect endogenous defense capacity [15,16]. Recent reviews highlight not only these classical markers but also novel biomarkers—such as F2-isoprostanes and composite oxidative balance scores (OBS)—that provide a more comprehensive assessment of residual oxidative risk in hypertension, including in well-controlled cases [17,18,19]. Recent epidemiological analyses have demonstrated that individuals with higher OBS, representing a more favorable antioxidant/pro-oxidant balance, exhibit significantly reduced all-cause and cardiovascular mortality, even among those with well-controlled hypertension [20,21]. These findings suggest that residual oxidative stress, inadequately captured by standard macrovascular indices or inflammatory markers, remains clinically relevant for long-term risk stratification. However, it remains unclear whether a gradient of oxidative stress exists within this well-controlled hypertensive population, and how it relates to clinical characteristics such as age or BP levels at the upper end of the therapeutic range.

Given this knowledge gap, we hypothesized that individuals with well-controlled hypertension would demonstrate persistent oxidative stress compared to normotensive controls. Consequently, the primary objective of this pilot study was to compare key biomarkers of oxidative stress between these two groups. As a secondary exploratory objective, we investigated the association between these biomarkers and blood pressure levels, including a subgroup analysis to explore characteristics associated with high versus low oxidative stress.

## 2. Materials and Methods

### 2.1. Study Designs and Population

This study protocol was permitted by the Khon Kaen University Ethics Committee (HE671196) on 10 April 2024. This cross-sectional, observational pilot study was conducted at the outpatient department of Srinagarind Hospital, Faculty of Medicine, Khon Kaen University, Thailand, between May and December 2024. Prior to enrollment, all individuals gave their written informed permission.

Adults aged 18 to 70 years were eligible to participate in the study, which was conducted via sequential sampling. The subjects were divided into two groups: The normotensive control group (*n* = 20) included individuals with office BP < 130/80 mmHg on two separate occasions, with no history of hypertension or antihypertensive medication use. The well-controlled hypertensive group initially consisted of 20 patients. Six were subsequently excluded from the final analysis because their systolic blood pressures exceeded the inclusion criterion of <140 mmHg, resulting in a final cohort of *n* = 14. Inclusion criteria for hypertensive patients were a clinical diagnosis of hypertension for ≥6 months, stable antihypertensive therapy for ≥3 months, consistent office BP < 140/90 mmHg, and self-reported medication adherence >80%. Exclusion criteria comprised a history of cardiovascular disease (myocardial infarction, stroke), diabetes mellitus (DM), chronic kidney disease (eGFR < 60 mL/min/1.73 m^2^), active inflammatory or autoimmune disorders, malignancy or terminal illness, pregnancy or lactation, current smoking or recent smoking cessation (<6 months), use of antioxidant supplements within 4 weeks, or inability to provide informed consent.

### 2.2. Clinical Measurements

Standardized questionnaires were used to obtain demographic and medical history information. Body mass index (BMI) was calculated using anthropometric data such as height and weight. Blood pressure and heart rate were monitored after 15 min of rest in a temperature-controlled environment (~25 °C) using a certified oscillometric instrument (ROSSMAX CF155f, Taiwan, China). Three BP readings were obtained at two-minute intervals, and the mean of the last two was used for analysis.

### 2.3. Ankle–Brachial Index (ABI)

A sphygmomanometer (Omron Healthcare Co., Ltd., Kyoto, Japan) was used to evaluate ABI using a standard technique. SBP was taken from both brachial arteries and both ankles (dorsalis pedis and posterior tibial arteries). The ABI is the ratio of the highest ankle systolic pressure to the maximum brachial systolic pressure; values between 0.9 and 1.4 were considered normal.

### 2.4. Laboratory Assays

After an overnight fast of at least 12 h, venous blood samples were taken into EDTA tubes. Biochemical assays, including fasting glucose, HbA1c, lipid profile (total cholesterol, triglycerides, HDL, LDL), and serum creatinine, were carried out using automated laboratory methods. eGFR was estimated with the CKD-EPI equation. Plasma was separated by centrifugation (3000 rpm, 15 min, 4 °C) and kept at −80 °C until analysis.

Plasma MDA (primary endpoint) was measured using a commercial TBARS assay as previously described [22]. SOD activity was detected using an ELISA kit (Dojindo Laboratories, Kumamoto, Japan), and catalase activity was measured spectrophotometrically according to standard protocols. Inflammatory markers (TNF-α, IL-6) were determined using high-sensitivity ELISA kits (Reed Biotech Ltd., Wuhan, China; Abcam, Cambridge, UK). All assays were conducted in duplicate by laboratory workers unaware of group assignment. The inter- and intra-assay coefficients of variance were below 10% in all assays. Due to insufficient sample volume, final analyses for inflammatory markers were performed on 19 normotensive and 13 hypertensive participants.

### 2.5. Statistical Analysis

As a pilot study, the sample size (*n* = 34) provides adequate power (>80%) to reveal large effect sizes (Cohen’s d ≥ 0.8), consistent with exploratory biomarker studies. Statistical analyses were conducted utilizing GraphPad Prism version 10.4.2 (GraphPad Software, San Diego, CA, USA). The Shapiro–Wilk test evaluated the normality of the data. Normally distributed continuous variables are expressed as mean ± standard deviation (SD) and analyzed using independent t-tests; Welch’s t-test was employed when variances were uneven. Non-normally distributed data are represented as median (interquartile range [IQR]) and analyzed using the Mann–Whitney U test. Categorical variables are expressed as counts (*n*) and percentages (%), examined by Chi-square or Fisher’s exact test as suitable. Spearman’s rank correlation coefficients were computed to evaluate the relationship between SBP and MDA levels. All tests were bilateral, and *p* < 0.05 was deemed statistically significant.

## 3. Results

### 3.1. Baseline and Hemodynamic Characteristics

A total of 34 participants—20 normotensive individuals and 14 patients with well-controlled hypertension—were included in the final analysis. Baseline demographic, anthropometric, and clinical characteristics are summarized in Table 1. The two groups were well-matched for age, sex, and BMI (all *p* > 0.05). Hypertensive patients had mean SBP and DBP not significantly different from normotensive controls (SBP: 126.50 ± 13.02 vs. 119.30 ± 12.56 mmHg, *p* = 0.12; DBP: 76.95 ± 7.12 vs. 74.62 ± 7.23 mmHg, *p* = 0.36). Resting heart rates did not differ significantly (*p* = 0.33). Antihypertensive medication use among hypertensive patients was as follows: ACE inhibitor (*n* = 3, 21.4%), ARB (*n* = 3, 21.4%), CCB (*n* = 4, 28.6%), and a combination of ARB and CCB (*n* = 4, 28.6%).

### 3.2. ABI, Biochemical, and Inflammatory Parameters

**Figure 1:**
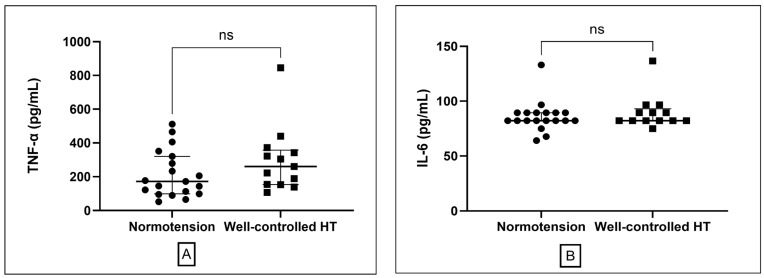
Comparison of inflammatory markers between normotensive and well-controlled hypertensive groups. (**A**) Plasma tumor necrosis factor-alpha (TNF-α) levels presented as median and interquartile range. (**B**) interleukin-6 (IL-6) presented as median and interquartile range. ns; non-significant different.

Vascular function (ABI) was comparable between groups for both left and right legs (*p* > 0.05 for both; Table 1). Metabolic parameters, including fasting plasma glucose (FPG), lipid profile (TC, TG, HDL, LDL), and renal function (serum creatinine, eGFR), did not differ significantly (all *p* > 0.05). circulating inflammatory markers, including TNF-α and IL-6, were also similar between normotensive (*n* = 19) and hypertensive participants (*n* = 13) (*p* = 0.15 and *p* = 0.51, respectively; Table 1, Figure 1).

### 3.3. Oxidative Stress Markers

**Figure 2:**
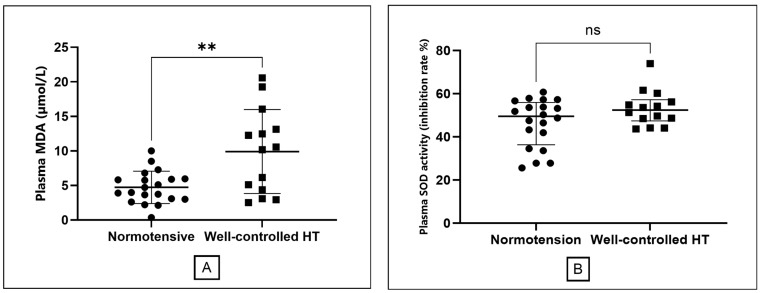
Comparison of oxidative stress markers between the normotensive and well-controlled hypertensive groups. (**A**) Plasma malondialdehyde (MDA) levels, presented as mean ± standard deviation. (**B**) Superoxide dismutase (SOD) activity presented as median with interquartile range. The double asterisk (**) indicates a statistically significant difference (*p* = 0.008) for MDA. ns; non-significant different.

The primary endpoint, MDA concentrations, was significantly elevated in the well-controlled hypertension group compared to normotensive controls (9.91 ± 6.07 vs. 4.73 ± 2.34 µmol/L; *p* = 0.008; Table 1, Figure 2A). No significant difference was observed in SOD activity between groups [hypertensive: 52.46 (47.41–57.23) U/mL; normotensive: 49.55 (36.40–56.00) U/mL; *p* = 0.19; Figure 2B]. There was a trend toward higher catalase activity in the hypertensive group [27.64 (22.84–29.72) U/mL] compared to controls [23.99 (20.88–26.87) U/mL]; nonetheless, this difference did not attain statistical significance (*p* = 0.10).

### 3.4. Subgroup Analysis of Oxidative Stress in the Hypertensive Group

To further explore the characteristics of patients with persistent oxidative stress, we stratified the well-controlled hypertensive group (*n* = 14) into High MDA and Low MDA subgroups based on the median MDA value (8.16 µmol/L). As shown in Table 2, the High MDA group was significantly older than the Low MDA group (median age 63 vs. 53 years, *p* = 0.03). Furthermore, there was a strong trend towards higher systolic blood pressure in the High MDA group, with levels approaching the upper limit of the controlled range (median SBP 137.0 vs. 120.7 mmHg, *p* = 0.051). No notable variations were detected in medication distribution, BMI, metabolic parameters, or inflammatory markers between the two groupings.

### 3.5. Correlation Analysis

**Figure 3:**
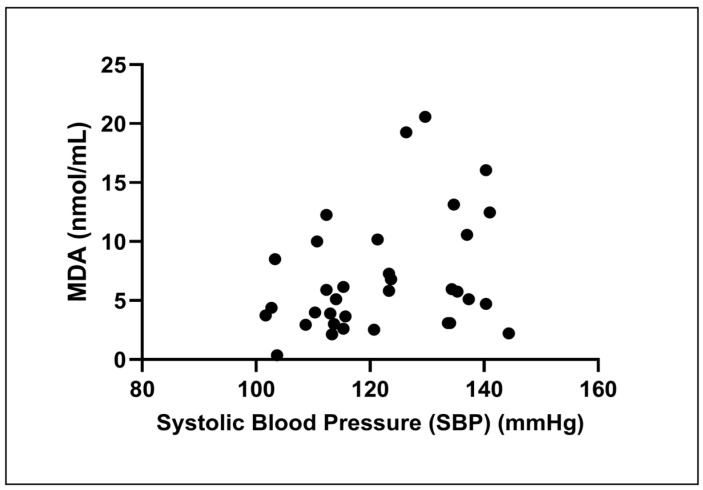
Correlation between systolic blood pressure (SBP) and malondialdehyde (MDA) levels. The scatter plot illustrates the relationship in all participants (*n* = 34). A Spearman correlation analysis revealed a weak, non-significant positive correlation between the two variables (r_s_ = 0.29, *p* = 0.09).

The Spearman correlation analysis found a slight, non-significant positive association between SBP and MDA levels (r_s_ = 0.29, *p* = 0.09; Figure 3).

## 4. Discussion

The principal finding of this pilot study is that adults with well-controlled hypertension exhibit persistent systemic oxidative stress—measured by MDA concentrations—even after achieving guideline-recommended blood pressure targets and in the absence of macrovascular or inflammatory abnormalities. This observation is consistent with contemporary evidence that cardiovascular risk in hypertension extends beyond hemodynamic normalization, implicating additional pathophysiologic processes such as enduring oxidative burden and vascular remodeling [5,15,23].

Elevated MDA, a key product of lipid peroxidation and robust redox biomarker, is mechanistically linked to impaired nitric oxide signaling, endothelial dysfunction, and progressive vascular injury—all recognized contributors to hypertension-related atherosclerosis and cardiovascular morbidity [15,24,25]. In well-controlled hypertension, inflammatory markers, including TNF-α and IL-6, were comparable to those in normotensive subjects, suggesting that effective blood pressure management may normalize systemic inflammatory activity. In addition, there is evidence to show that antihypertensive drugs, including ACE inhibitor [26], ARB [27] and CCB [28] may exhibit anti-inflammatory effects. Our findings reinforce reports that traditional indices such as ABI and inflammatory markers (TNF-α, IL-6) may not fully capture this persistent metabolic and vascular risk in hypertensive populations [13,29]. The absence of correlation between SBP and MDA observed in our study further suggests that oxidative stress may reflect pathophysiological processes independent of current BP measurements, a notion our subsequent subgroup analysis further clarified by revealing a more nuanced relationship within our patient cohort.

Our novel subgroup analysis provides deeper insight into this complexity. We found that hypertensive participants in the High-MDA group were significantly older (*p* = 0.03) and exhibited a strong trend towards higher systolic BP (*p* = 0.05) compared to their Low-MDA counterparts. The link with age is well-established, often attributed to declines in endogenous antioxidants and a chronic low-grade inflammatory state termed inflammaging [30,31]. Perhaps more clinically relevant, our finding that SBP at the upper end of the controlled range (130–140 mmHg) is associated with higher MDA challenges the notion of a benign binary BP threshold. It supports the concept of a continuous vascular risk spectrum and underscores the potential need for more individualized risk stratification, even in patients considered well-controlled [6].

Regarding antioxidant defenses, we observed a trend toward higher catalase activity in the hypertensive group (*p* = 0.10). This may reflect a partial but ultimately insufficient compensatory upregulation in response to the significantly increased oxidative burden [15,16,32]. The inadequacy of this response is evidenced by the significantly elevated MDA levels, confirming a net pro-oxidant state. This finding underscores the importance of assessing both pro-oxidant markers (like MDA) and antioxidant capacity to obtain a complete picture of the overall redox balance in hypertensive patients [21].

Notably, the normal ABI values observed across our cohort, even within the high-MDA group, are striking. This finding strongly suggests that biochemical markers like MDA can detect early vascular dysfunction long before it manifests as the macrovascular changes identified by ABI. Our study therefore supports a growing consensus calling for the integration of broader redox biomarker panels—such as F2-isoprostanes or OBS—to improve risk stratification in patients whose residual vascular risk is underestimated by standard clinical measures [17,25]. From a clinical perspective, our data highlight that oxidative stress is a key residual cardiovascular risk factor in hypertensive patients, even when BP is well-controlled. This implies that routine risk assessment could be enhanced by incorporating redox biomarkers to identify these high-risk individuals. Looking forward, while preclinical evidence suggests that antioxidant or lifestyle interventions could mitigate this residual risk, their clinical efficacy must be confirmed in rigorously designed human trials [33,34].

Several limitations of this study should be acknowledged. First, the cross-sectional design precludes causal inference, and the modest sample size limits the statistical power and generalizability of our findings. Second, our reliance on a single TBARS-based MDA assay, while common, is susceptible to specific issues; future studies would benefit from incorporating more robust markers like F2-isoprostanes. Furthermore, our univariate analyses did not account for potential confounders such as comorbidities, medication regimens, or lifestyle factors. Finally, ABI reflects structural rather than functional vascular health; including functional measures like flow-mediated dilation or pulse wave velocity would provide a more comprehensive vascular assessment in future research

Despite these limitations, this study provides valuable preliminary evidence that systemic oxidative stress persists even in adults with well-controlled hypertension. To build upon these findings, future research should employ larger, longitudinal designs with multi-biomarker protocols and functional vascular assessments. Ultimately, randomized controlled trials of targeted antioxidant or lifestyle interventions are necessary to confirm clinical benefits and inform future risk-based management strategies in hypertension.

## 5. Conclusions

In conclusion, residual oxidative stress—as reflected by increased plasma MDA—persists in patients with well-controlled hypertension. While this oxidative state appears broadly independent of BP when viewed as a whole, it is notably more pronounced in older patients and in those with systolic BP approaching the upper limit of the controlled range. These findings support the need for comprehensive, biomarker-based risk assessment and further investigation into targeted strategies for mitigating persistent redox imbalance in hypertension.

## Figures and Tables

**Table 1:** Baseline characteristics, hemodynamic, and biochemical parameters of normotensive and well-controlled hypertensive adult.

Variable	Normotension (*n* = 20)	Well Controlled Hypertension (*n* = 14)	*p* Value
**Demographic and anthropometric data**			
Age, years	57.80 ± 6.06	60.14 ± 5.70	0.26
Female sex, n (%)	16 (80.00)	9 (64.30)	0.34
BMI, kg/m^2^	24.88 ± 2.61	24.15 ± 2.31	0.40
**Antihypertensive medication, n (%) ^a^**			
ACE inhibitor	0 (0)	3 (21.40)	-
ARB	0 (0)	3 (21.40)	-
CCB	0 (0)	4 (28.60)	-
ARB + CCB	0 (0)	4 (28.60)	-
**Hemodynamic parameters**			
SBP, mmHg	119.30 ± 12.56	126.50 ± 13.02	0.12
DBP, mmHg	74.62 ± 7.23	76.95 ± 7.12	0.36
Resting heart rate, beats/min	70.10 ± 10.46	66.74 ± 9.23	0.33
**ABI parameters**			
Left ABI	1.10 ± 0.06	1.09 ± 0.05	0.65
Right ABI	1.11 ± 0.06	1.09 ± 0.05	0.22
**Biochemical parameters**			
FPG, mg/dL	90.95 ± 8.65	90.64 ± 9.25	0.92
TC, mg/dL	194.60 ± 25.90	181.80 ± 22.02	0.11
TG, mg/dL	108.00 ± 49.70	95.21 ± 34.78	0.39
HDL, mg/dL	59.95 ± 8.33	59.21 ± 10.72	0.83
LDL, mg/dL	117.30 ± 24.00	108.40 ± 22.63	0.28
Creatinine, mg/dL	0.83 ± 0.16	0.910 ± 0.18	0.19
eGFR, mL/min/1.73 m^2^	85.53 ± 12.66	78.40 ± 16.16	0.18
**Inflammatory markers ^b^**			
TNF-α, pg/mL ^c^	172.10(98.40–320.70)	260.1 (153.8–357.30)	0.15
IL-6, pg/mL ^c^	82.18 (82.18–89.45)	82.18 (82.18–93.09)	0.51
**Oxidative stress markers**			
MDA, µmol/L	4.73 ± 2.34	9.91 ± 6.07	0.008
SOD, U/mL ^b^	49.55 (36.40–56.00)	52.46 (47.41–57.23)	0.19
Catalase, U/mL ^b^	23.99 (20.88–26.87)	27.64 (22.84–29.72)	0.10

Data are expressed as mean ± standard deviation (SD), median (interquartile range [IQR]), or *n* (%). Statistically significant *p*-values (*p* < 0.05) are shown in bold. The independent t-test, Welch’s t-test, or Mann–Whitney U test, as applicable, were used to compare continuous variables. Fisher’s exact test or the Chi-square test were used to compare categorical variables. ^a^ *p*-value not calculated as no antihypertensive medications were used in the normotensive group. ^b^ non-normally distributed data; presented as median (IQR). ^c^ For inflammatory markers, sample size was *n* = 19 for normotensive and *n* = 13 for hypertensive group due to missing values.

**Table 2:** Baseline Characteristics of Study Participants Stratified by MDA Level.

Characteristics	Low MDA (MDA < 8.16) (*n* = 7)	High MDA (MDA ≥ 8.16) (*n* = 7)	*p* Value
**Demographic**			
Age, years	53 (53–62)	63 (61–65)	0.03
**Anthropometric**			
BMI, kg/m^2^	23.32 (20.75–25.40)	25.19 (24.03–26.75)	0.16
**Antihypertensive medication, n (%) ^a^**			
ACE inhibitor	2 (28.60)	1 (14.30)	0.53
ARB	2 (28.60)	1 (14.30)	0.53
CCB	2 (28.60)	2 (28.60)	1.00
ARB + CCB	1 (14.30)	3 (42.90)	0.27
**Hemodynamic parameters**			
SBP, mmHg	120.70 (108.7–133.7)	137.0 (126.3–139.7)	0.05
DBP, mmHg	78.33 (68.0–79.67)	75.33 (71.33–86.0)	>0.99
Resting heart rate, beats/min	68.67 (59.67–76.33)	63.33 (59.67–75.33)	0.74
**ABI parameters**			
Left ABI	1.10 (1.06–1.20)	1.10 (1.02–1.15)	>0.99
Right ABI	1.09 (1.06–1.14)	1.06 (1.05–1.09)	0.33
**Biochemical parameters**			
FPG, mg/dL	87.0 (85.0–95.0)	92.0 (86.0–94.0)	0.69
HbA1c, %	5.9 (5.50–6.0)	5.6 (5.13–6.08)	0.48
TC, mg/dL	183.0 (166.0–213.0)	181.0 (163.0–185.0)	0.48
TG, mg/dL	81.0 (70.0–125.0)	101.0 (65.0–111.0)	0.88
HDL, mg/dL	57.0 (54.0–69.0)	56.0 (54.0–63.0)	0.65
LDL, mg/dL	98.0 (96.0–137.0)	97.0 (87.0–129.0)	0.64
Creatinine, mg/dL	0.93 (0.67–1.19)	0.92 (0.81–1.06)	0.77
eGFR, mL/min/1.73 m^2^	75.30 (69.81–102.1)	74.30 (66.95–92.51)	0.90
**Inflammatory markers ^b^**			
TNF-α, pg/mL	260.10 (152.70–321.80)	282.40 (151.0–389.0)	0.73
IL-6, pg/mL	82.18 (82.18–96.73)	85.82 (82.18–91.27)	0.96
**Oxidative stress markers**			
MDA, µmol/L	4.40 (2.94–6.15)	13.13 (12.26–19.26)	<0.001
SOD, U/mL	49.70 (48.49–53.61)	56.23 (44.08–61.65)	0.26
Catalase, U/mL	29.05 (22.60–30.21)	26.62 (23.03–28.41)	0.20

For continuous variables, the data are shown as the median (interquartile range), and for categorical variables, the number (%). ^a^ *p*-values calculated using Fisher’s exact test ^b^ Inflammatory markers data available for *n* = 7 in Low MDA group and *n* = 6 in High MDA group *p*-values for continuous variables calculated using Mann–Whitney U test *p* < 0.05. ACE, angiotensin-converting enzyme; ARB, angiotensin II receptor blocker; BP, blood pressure; CCB, calcium channel blocker; eGFR, estimated glomerular filtration rate; HbA1c, glycated hemoglobin; HDL, high-density lipoprotein; IL-6, interleukin-6; LDL, low-density lipoprotein; MDA, malondialdehyde; TNF-α, tumor necrosis factor-alpha.

## Data Availability

The original contributions presented in this study are included in the article/Supplementary Materials. Further inquiries can be directed to the corresponding author.

## Supplementary Materials

The following supporting information can be downloaded at: https://www.mdpi.com/article/10.3390/medsci13040292/s1, Figure S1: Correlation between diastolic blood pressure (DBP) and malondialdehyde (MDA) levels.

## Abbreviations

The following abbreviations are used in this manuscript:

ACE	Angiotensin-converting enzyme inhibitor
ABI	Ankle–brachial index
ARB	Angiotensin receptor blocker
BMI	Body mass index
CCB	Calcium channel blocker
DBP	Diastolic blood pressure
eGFR	Estimated glomerular filtration rate
FPG	Fasting plasma glucose
HbA1c	Glycated hemoglobin
HDL	High-density lipoprotein
IL-6	Interleukin-6
LDL	Low-density lipoprotein
MDA	Malondialdehyde
SBP	Systolic blood pressure
SOD	Superoxide dismutase
TC	Total cholesterol
TG	Triglycerides
TNF-α	Tumor necrosis factor-alpha
OBS	Oxidative balance scores
