# Supplementary material for: Assessment of Residual Oxidative Stress in Patients with Well-Controlled Hypertension: A Pilot Cross-Sectional Study
Source: Med Sci (Basel). 2025 Nov 28;13(4):292. doi: 10.3390/medsci13040292 (PMC12734460; doi:10.3390/medsci13040292)
Supplement: Supplementary file 1 [file medsci-13-00292-s001.zip › medsci-3955480-supplementary.pdf]

Supplementary Figure

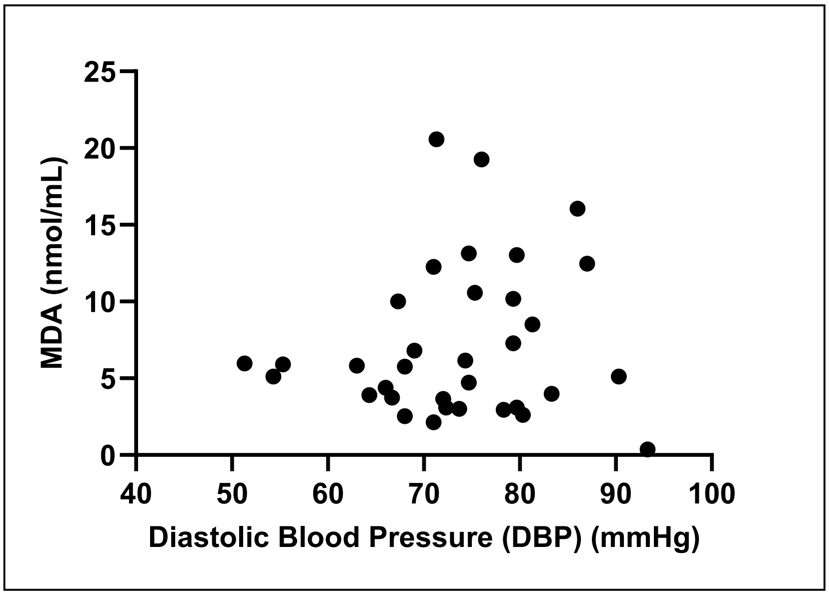

Figure S1. Correlation between diastolic blood pressure (DBP) and malondialdehyde (MDA) levels. The scatter plot illustrates the relationship in all participants ($n = 34$). A Spearman correlation analysis revealed a weak, non-significant correlation between the two variables (rs = 0.093, $p = 0.60$).
